# Shaker potassium channel mediates an age-sensitive neurocardiac axis regulating sleep and cardiac function in Drosophila

**DOI:** 10.21203/rs.3.rs-6616119/v1

**Published:** 2025-05-19

**Authors:** Kishore Madamanchi, Dalton Bannister, Ariel Docuyanan, Shruti Bhide, Girish Melkani

## Abstract

The *Shaker* (Sh) gene in *Drosophila melanogaster* encodes a voltage-gated potassium channel essential for regulating neuronal excitability and cardiac function. While Sh's role in neuronal physiology, particularly in sleep regulation, is relatively well-studied, its contribution to cardiac physiology and inter-tissue communication remains poorly understood. This study explores the impact of *Sh* mutations ( *Shmns* and *Sh5* ) on heart function and sleep/circadian behaviors, aiming to uncover potential neurocardiac interactions in an age-dependent manner. Cardiac performance and locomotor/sleep activity were assessed in mutant and control flies across aging cohorts under both normal and circadian-disrupted conditions, with and without time-restricted feeding (TRF). *Shmns* mutants displayed progressive, age-dependent cardiac dysfunction, including increased heart period, elevated arrhythmicity index, prolonged systolic and diastolic intervals, and diminished heart rate and fractional shortening, as well as disorganization of actin-containing myofibrils. These defects were paralleled by severe sleep loss and hyperactivity, suggesting a strong link between sleep/circadian dysregulation and cardiac impairment. Circadian disruption further exacerbated both cardiac and behavioral phenotypes, whereas TRF partially ameliorated these defects, highlighting a modulatory role for feeding timing. Tissue-specific knockdowns of *Sh* in cardiac and neuronal tissues recapitulated both heart and sleep abnormalities, with neuronal knockdown alone significantly impairing cardiac function, supporting a neurocardiac regulatory axis. Altogether, our findings reveal that Shaker channels mediate a critical, age-sensitive interplay between sleep/circadian systems and cardiac homeostasis in *Drosophila* . This work provides mechanistic insight into neurocardiac communication and suggests that *KCNA1* -linked human channelopathies may similarly impact sleep and cardiovascular health, offering a potential translational framework for age-related disorders.

## Introduction

The *Shaker* gene (*Sh*) in *Drosophila melanogaster* was initially identified based on the distinguishing leg-shaking phenotype displayed by mutant flies under ether anesthesia (Kaplan and Trout 1969; Kim and Nimigean 2016). This gene encodes the alpha subunit of the voltage-gated potassium channel (Kv), essential for regulating neuronal excitability by mediating membrane repolarization after action potentials (Papazian et al. 1987; Tempel et al. 1987). They are primarily expressed in the axons and synaptic terminals of the *Drosophila* nerves (Cirelli et al. 2005a). Which mediates a rapidly inactivating A-type current through an N-terminal ball and chain mechanism (Pongs et al. 1988). In humans, the homologs of the *Shaker* gene belong to potassium voltage-gated channel subfamily A member 1 (*Kcna1*). Some voltage-gated potassium channels, like Shaker and the mammalian homologs Kv1, are primarily expressed in the brain, more specifically in regions of the axons and synaptic terminals, and the heart, hence influencing the function of both organs simultaneously (Glasscock 2019a). Channelopathies in these tissues can lead to both neurologic and cardiac dysfunction either independently or in connection with each other.

The KV1 channel, which is encoded by the *Kcna1* gene, has been studied extensively in mice (Tempel et al. 1988). *Kcna1* gene knockout mice display aberrant neuronal discharge and seizures, which are like the leg shaking that *Drosophila Shaker* mutants display under ether anesthesia (Ueda and Wu 2006). *Shaker* mutants show similar aberrant signaling in motor neurons and increased neuromuscular junction transmission (Ueda and Wu 2006). The *Shaker* gene codes for the alpha subunit of a tetrameric voltage-gated K-channel. For each subunit, domains S1-S4 are the voltage sensor, and S5-S6 form the pore (Whicher and MacKinnon 2016). Mutations in *Shaker* significantly disrupt potassium currents, influencing neuronal firing patterns, synaptic plasticity, and various neurological functions (Kim et al. 2020). These disruptions impact essential behaviors, including locomotion, circadian rhythmicity, sleep regulation, and overall lifespan (Cirelli et al. 2005b; Koh et al. 2008a; Flourakis et al. 2015). In humans, the homolog of *Drosophila Shaker* is the *Kcna* gene family, especially *Kcna1*. The *Kcna1* encodes voltage-gated potassium channel subunits involved in neuronal excitability and signal transduction. Mutations in *Kcna1* are linked to neurological disorders such as episodic ataxia type 1 (EA1) (D'Adamo et al. 2015), featuring muscle spasms, episodic loss of motor control, tremors, and sleep abnormalities, highlighting the evolutionary conservation of potassium channel functionality between flies and humans (Paulhus et al. 2020). The *Shaker* gene is found on the X chromosome and is recessive. *Drosophila* wild type and female heterozygotes sleep 9–15 hours/day, whereas male and female homozygotes sleep 4–5 hours/day (Cirelli et al. 2005c).

Among *Shaker* mutants, mini sleep (*mns*) and *Shaker-5* (*Sh5*) have been extensively characterized. The mini sleep mutant, characterized by substantially reduced sleep duration, provides a robust model for studying insomnia-like behaviors and their broader physiological consequences, including stress response and metabolic alterations (Bringmann 2019). The *Sh5* mutant, lacking functional Shaker channels, exhibits severe electrophysiological defects and altered behavior, emphasizing the integral role of potassium channels in maintaining neuronal stability and healthy aging (Bushey et al. 2007). Recent studies underscore the significance of Shaker channel dysfunction in aging-related pathologies, linking aberrant neuronal activity to cardiovascular decline, sleep disturbances, and reduced lifespan in *Drosophila* (Bushey et al. 2007; Koh et al. 2008b). The targeted expression of *Shaker* mutations in specific tissues, such as the neuronal and heart, offers an effective approach to dissecting their individual and combined roles in cardiac and circadian involvement.

One of the goals in our lab is to find genes that link sleep with the manifestation of cardiovascular disease (Guo et al. 2024a). We came across the *Shmns* mutant and discovered that these flies had a novel phenotype consisting of cardiac dysrhythmia. The cardiovascular function of *Drosophila Shaker* mutants has not previously been studied, so we decided to further investigate whether there is a relationship between the short sleeping phenotype and the novel cardiac abnormality. We sought to determine the source of the cardiac phenotype and whether the Shaker channel had neurocardiac function like its homolog Kv1.1. The heart of the fruit fly has extensive neuronal innervation and glutamatergic neuromuscular junctions (Dulcis and Levine 2003, 2005a). Knowing that the *Shaker* gene is expressed in both the brain and the heart, we want to determine if the Shaker channel has independent roles in the heart and brain or if it has a neurocardiac role like its mammalian homolog Kv1.1 (Glasscock

2019b). We also wish to determine if our well-studied method of time-restricted feeding will ameliorate the cardiac arrhythmia in *Shmns* flies and whether it will affect the amount of sleep (Guo et al. 2024b).

## Results

2.1. *Shaker Shmns* mutant allele led to severely compromised cardiac physiology, whereas *Sh5* mutant allele has a subtle impact on cardiac performance in age age-dependent manner in both male and female flies:

*Shakermns* is a specific mutant allele of the *Shaker* gene responsible for shortened sleep duration. These flies need significantly less sleep compared to wild-type flies. This occurs because $K^+$ channels play a critical role in regulating neuronal excitability, and their dysfunction leads to hyperactivity and reduced sleep (Kim et al. 2020). The heart physiology and sleep/activity of flies were analyzed for the *Shmns* and *Sh5* and compared to that of the age and sex-matched *Drosophila* control line $w^{1118}$. Different variables of heart function were measured and represented in individual graphs: A. Heart rate (HR) B. Heart period (HP), C. Arrhythmicity index (AI), D, E. Diastolic (DI) and Systolic intervals (SI), F, G. Diastolic diameter (DD) and Systolic diameter (SD), and H. Fractional shortening (FS). (Figure. 1A-H) represents data collected from three-week and five-week-old male flies. *Shmns* showed an increase in HP, AI, DI, and SI than $w^{1118}$ and *Sh5*, as well as significantly decreased FS and HR compared to $w^{1118}$. *Sh5* shows a significant reduction in FS and an increase in HR compared to $w^{1118}$. DD and SD did not find any significant difference between the control and mutants. The *Shmns* five-week flies showed an increased HP, DI, and SI than $w^{1118,}$ and *Sh5* and AI, DD, and SD showed no change, but FS was significantly reduced compared to $w^{1118}$ in both *Shmns* and *Sh5*. The HR was reduced in *Shmns* compared to $w^{1118}$ and *Sh5*. Whereas *Sh5* showed reduced SI, DD, and FS compared to *Sh5* OC and $w^{1118}$. (Figure. 1I-J). Phalloidin staining of the heart showed a significant difference between $w^{1118}$ and *Shmns* heart muscle density, where *Shmns* heart was found with reduced myofibril percentage than the control $w^{1118}$. (Figure. 1K-P), showed sleep/activity analysis $w^{1118}$ and *Shmns*, with significantly increased activity and reduced sleep in *Shmns* compared to $w^{1118}$ for three weeks and five weeks of age. *Sh*$^{mns}$ severely impairs cardiac function and reduces sleep, with age-related worsening in both sexes. *Sh5* shows milder, age-dependent cardiac effects. These results highlight Shaker $K^+$ channels' critical role in regulating heart function and sleep.

2.2. Trans heterozygous (*Shmns/+: Sh5/+Shaker* mutants do not aggravate cardiac physiology defect linked with the *Shmns* mutant:

We have investigated the impact of both *Shmns* and *Sh*5 alleles on female flies. (A-H) represents cardiac data collected from three and five-week-old females. *Shmns* showed a significant increase in HP, DI, and SI compared to controls and *Shmns*x *Sh5* flies. AI, DD in *Shmns*x *Sh5* than *Shmns* flies. *Sh5* showed an increase in HP and DI compared to $w^{1118}$, and with *Shmns* HP, SI. *Sh5* showed a significant difference with *Shmns* x *Sh5* in HP, DI, and SI. SD and FS did not show any significant difference among the groups.

At five weeks of age, only *Shmns* flies HP, AI, DI and SI increased than $w^{1118}$. *Sh5* flies AI, DI, SI, SD, and FS are reduced than *Shmns*. *Shmns*x *Sh5* flies showed decreased HP, AI, DI, and SI than *Shmns* flies, and reduced SD and increased FS compared to *Sh5* flies. The HR was reduced in *Shmns* flies at both ages, and there was no significant difference in *Shmns*x *Sh5* and *Sh5* flies, but *Shmns*x *Sh5* flies showed a significant increase than *Shmns* flies. Trans-heterozygous *Shmns/Sh*$^{5}$ mutants do not exacerbate *Shmns*-induced cardiac defects. Instead, they partially rescue heart function, showing improved parameters like HP, DI, SI, and FS compared to *Shmns* alone, suggesting a non-additive or compensatory genetic interaction between *Shaker* alleles.

## 2.3. Circadian disruption further deteriorates cardiac physiological and sleep/circadian dysregulation linked with *Shmns* mutant:

Light significantly impacts sleep-wake cycles and heart function. Since the *Shmns* mutant flies are genetically predisposed to have shorter sleep cycles compared to $w^{1118}$ flies, we performed heart physiology and sleep/activity analysis to understand the impact of light-light cycle changes in male flies. (Figure. 3A-H) represents the impact of light/dark (LD) and light/light (LL) cues-induced circadian cycle disruption on cardiac physiology and sleep/activity patterns of three-week-old flies. (A-H) HP, AI, DI, SI, DD, and SD did not show any statistically significant difference, but FS was reduced in LL than LD $w^{1118}$. Similarly, LD vs LL conditions did not show a statistically significant difference in heart parameters of *Shmns* flies. HP, AI ($p < 0.05$), DI, SI, DD, and SD showed a significant increase in LD *Shmns* than LD $w^{1118}$. HP, DI, and SI significantly increased in LL *Shmns* flies compared to LL $w^{1118}$, whereas FS was reduced. The HR was reduced in LD *Shmns* flies and LL *Shmns* flies compared to respective controls, suggesting the light-light cycles regulate heart functions differently in mutant flies than $w^{1118}$. In females (Fig. 3I-P) at 3 weeks HR, FS was reduced, and HP, AI significantly increased in LL compared to LD in $w^{1118}$ flies. In *Shmns* flies SD was reduced and FS increased significantly in LL compared to the LD condition. LD *Shmns* flies showed increased HP, DI, SI, DD, and reduced HR than LD $w^{1118}$. During LL *Shmns* flies showed increased HP, DI, SI, FS, and decreased DD and SD were observed compared to LL $w^{1118}$. Phalloidin staining (Figure. 3Q, R) indicates reduced organization of F-actin containing myofibrillar percent in heart muscles of LL $w^{1118}$ and LL *Shmns* than LD $w^{1118,}$ with no significant difference with other genotypes. We further checked the impact of heart physiology on sleep/activity parameters (Fig. 3S-X), we found day activity, night activity, and total activity were increased, and day sleep, night sleep, and total sleep were reduced in LD *Shmns* than LD $w^{1118}$ flies. During LL condition, day activity, night, and total activity increased, and sleep significantly decreased in LL *Shmns* flies than $w^{1118}$ flies at 3 weeks of age. We have not observed any significant difference between LL and LD $w^{1118}$ and *Shmns* flies, respectively. *Shmns* flies under LL conditions showed elevated HP, AI, reduced HR, and sleep, with increased activity levels compared to $w^{1118}$. These results indicate that disrupted light cues worsen

*Shmns*-associated physiological and behavioral impairments, highlighting the interaction between circadian regulation and Shaker channel function.

### 2.4. Time-restricted feeding altered cardiac physiology and sleep/activity dysregulation linked with *Shmns* mutant flies:

To understand the impact of feeding cues on heart physiology and sleep/activity, we further employed well-studied feeding patterns in our lab, i.e., time-restricted feeding (TRF) and ad libitum feeding (ALF) patterns on *Shmns* male and female flies for heart physiology (Figure. 4A-H) in males and (I-P) in females and sleep/activity analysis using male flies at 3 weeks of age (Figure. 4Q-V). The male heart physiology did not show a significant difference between ALF *Shmns* and TRF *Shmns* male flies. Whereas in female flies, HP, AI, and DI decreased, and HR was increased in TRF *Shmns* flies, and SI, DD, SD, and FS did not show a significant difference compared with ALF *Shmns* flies. In males (Q-S) activity was increased, and sleep was reduced significantly in TRF than in ALF *Shmns* flies. TRF partially rescues cardiac dysfunction and sleep/activity disruptions in *Shmns* mutant flies. TRF improved heart function in females and increased activity with reduced sleep, in males, indicating feeding timing modulates *Shaker*-linked physiological outcomes. Based on our observation in female flies, TRF improved cardiac parameters such as reduced HP, AI, and DI, and increased HR. In males, TRF led to elevated activity and reduced sleep without major cardiac changes. These findings suggest that feeding timing can partially rescue *Shaker*-linked cardiac and behavioral impairments through circadian-aligned interventions.

### 2.5. Cardiac-specific knockdown of the *Shaker* gene led to compromised cardiac function:

We also revealed the role of the *Shaker* gene using the Gal4-*UAS* expression system to understand cardiac cardiac-specific role using the Hand-Gal4 driver. Briefly, progeny of 3-week-old male and female Hand-Gal4 flies crossed with $w^{1118}$, *Shaker* RNAi (BL#53347, BL#31680) genes and empty vectors for 2nd (attP40) and 3rd (attP2) chromosomes as internal controls. Compared to the Hand/+ experimental control AI, SI, DD, and FS showed a significant decrease in BL#53347. Whereas BL#31680 showed a significant increase in HP, DI, and a decrease in HR, AI, FS, and DD compared to the Hand/+ control. Female flies at 3 weeks (Fig. 5I-P), BL#53347 showed a significant decrease in AI, DD, and SD, compared to Hand/+ controls. Whereas BL#31680 showed a significant increase in SI, compared to the $w^{1118}$ control. The HR was not showing any significant difference. Cardiac-specific knockdown of the *Shaker* gene using Hand-Gal4 disrupts heart function, with BL#53347 and BL#31680 showing altered intervals, diameters, and reduced FS. These results confirm a direct, cardiac-intrinsic role for *Shaker* in maintaining normal heart physiology. Based on observation of both BL#53347 and BL#31680 flies showing altered intervals, diameters, and reduced fractional shortening. These results demonstrate that *Shaker* function in cardiomyocytes is essential for maintaining proper cardiac rhythm, contraction strength, and structural integrity, underscoring its intrinsic role in *Drosophila* heart physiology.

### 2.6. Panneuronal knockdown of the *Shaker* gene led to altered cardiac physiology:

We have revealed the significance of the *Shaker* gene in a non-cell-autonomous manner upon its knockdown in neuronal cells using the Elav-Gal4 driver. Briefly, the progeny of 3-week-old male and female flies after crossing with the Elav-Gal4 driver with $w^{1118}$, *Shaker* RNAi (BL#53347, BL#31680) genes and empty vectors for 2nd (attp40) and 3rd (attP2) chromosomes as internal controls were used for cardiac physiology. As shown in Fig. 6. (A-H) Cardiac physiology of males. BL#53347 flies showed a significant decrease in AI, DD, and FS compared to $w^{1118}$, and BL#31680 flies showed a significant decrease in SI, AI, FS, and an increase in SD compared to $w^{1118}$, and HR did not show any significant difference. Figure 6 (I-P) represents female flies at 3 weeks of age. BL#53347 flies showed a significant decrease in AI and FS, and BL#31680 flies showed a significant decrease in HR and AI and a significant increase in HP, DI, and SI compared to $w^{1118}$. (Q-V) Sleep/activity analysis at 3 weeks showed no difference in activity, but day sleep, night sleep, and total sleep in BL#53347 were reduced compared to $w^{1118}$. Whereas we did not observe a significant change in BL#31680 in sleep/activity parameters. Both RNAi lines showed disrupted cardiac parameters, particularly reduced arrhythmicity index and fractional shortening. Additionally, *Shaker* knockdown via BL#53347 reduced sleep without affecting activity, highlighting the neural influence of *Shaker* on heart function and sleep regulation.

## Methods

*Drosophila Diet and Feeding-Fasting Regimens*:

Flies were reared on a standard laboratory diet consisting of 11 g/L agar, 30 g/L active dry yeast, 55 g/L yellow cornmeal, 72 mL/L molasses, 8 mL/L 10% nipagen, and 6 mL/L propionic acid. The flies were maintained at a controlled temperature of 25°C with 50% relative humidity under a 12-hour light/12-hour dark (LD) cycle. Newly eclosed $w^{1118}$ adults were collected and sex-separated into groups of 25–30 individuals on the third day post-eclosion. Feeding and light exposure regimens commenced on the seventh day. In the LD condition, flies experienced a 12-hour light/12-hour dark cycle, whereas those in the LL group were exposed to continuous light (12 hours light/12 hours light). Flies designated for ad libitum feeding (ALF) or time-restricted feeding (TRF) were provided access to standard diet vials beginning at zeitgeber time zero (ZT0), which corresponded to 8:00 AM (light onset). At 6:00 PM (light offset), ALF flies remained on their regular diet, while TRF flies were transferred to vials containing 1.1% agar. All experimental flies were transferred to fresh media every three days (Villanueva et al. 2019). To investigate the role of *Shakermns* and *Shaker5* alleles on sleep/activity and cardiac function, appropriate light and dietary interventions were implemented to evaluate their physiological impact on wild-type and mutant flies.

*Drosophila experimental stock*:

Fly stocks were ordered from the Bloomington *Drosophila* Stock Center and the Vienna *Drosophila* Resource Center. *Drosophila* experimental lines, including two *Shaker* mutants, *Shmns* (BDSC: 24259) and *Sh5* (BDSC: 111), and $w^{1118}$ for their control. Gal4-UAS RNAi lines used for the experiment included three lines for the *Shaker* gene (VDRC:104474, BDSC: 53347 & BDSC: 31680), two lines for controls (BDSC: 36303 & BDSC: 36304), and two drivers, one for heart-specific expression (Hand-Gal4) and another one for panneuronal expression (Elav-Gal4). The BDSC: 36303 control line carries an empty attP2 vector inserted in the 3rd chromosome, while the BDSC: 36304 control line carries an empty attP40 vector in the 2nd chromosome. The RNAi system uses small interfering RNA (siRNA) strands that are complementary to a gene of interest to experimentally silence its expression. These siRNA fragments do this by binding to the mRNA of the target gene and signaling for its degradation, thereby preventing its translation (Kim and Rossi, 2008). As previously reported (Guo et al. 2024c), flies from each UAS-RNAi *Shaker* and control fly line were crossed with virgin female flies from the Hand-Gal4 or Elav-Gal4 lines. Each of these control lines was crossed with Hand-Gal4 and Elav-Gal4 and compared to the *Shaker* RNAi lines that utilized the same vector and chromosome insertion site. These controls ensure that any possible phenotypes caused by the drivers, vectors, or chromosome insertions are accounted for and not mistakenly attributed to the silencing of the gene of interest.

*Fly aging*

The immediate progeny of each experimental and control cross was collected and gender separated. They were aged at 25$^{o}$C by replacing the food every 3–4 days (Villanueva et al. 2019; Guo et al. 2024c). Flies were dissected for 3 weeks and 5 weeks after eclosion, along with age-matched controls.

*Cardiac physiology*

Progeny from each line was collected at 3 weeks or 5 weeks of age for heart physiology analysis. Semi-intact heart dissections were performed to make the heart visible for recording (Fink et al. 2009a; Guo et al. 2024d). Flies were made unconscious with $CO_2$ gas and fixed on their backs to petri dishes with petroleum jelly. Under a light microscope, the head and thorax were removed, followed by a small incision at the apex of the abdomen. Plates were then flooded with warmed, aerated artificial hemolymph (physiological saline/trehalose/sucrose) to ensure hearts maintained myogenic activity during recording. The top of the abdomen was then removed, followed by the intestine and fat, exposing the heart. After the aeration of the petri dish, recordings were taken using an immersion microscope lens with an attached high-speed camera. Heart physiology was recorded between the second and third body segments to ensure consistency across flies. Recordings were made using a Promon u750 microscope camera in B&W at 200 frames/sec, for 30 seconds (Fink et al. 2009b). Videos were analyzed using semi-automated heart analysis (SOHA) software and data output was organized in excel files with M-mode records (Cammarato et al. 2015; Gill et al. 2015). The SOHA data we analyzed included heart period, arrhythmicity index, systolic and diastolic intervals, systolic and diastolic diameters and fractional shortening (Guo et al. 2024d). Using Prism 10 software, the SOHA output variables for experimental lines were statistically compared to the control lines using one-way analysis of variance (ANOVA). Significance was set at $p < 0.05$, and Tukey's multiple comparisons test was used for each data set.

*Cytological analysis*:

The fly bodies were maintained for 20 minutes in a 4% paraformaldehyde (PFA) solution after the heads, legs, and wings were removed for the cytological test (Guo et al. 2024c; Abou Daya et al. 2025). The fixed samples were then incubated for 15 minutes between each of the three PBS washes. The thoraces were longitudinally oriented in a cryomold using OCT (Fisher Scientific #4585) and flash-frozen on dry ice. Following cryosectioning to reach a thickness of 30 μm, three further washes in 1× PBS were carried out, each requiring a 15-minute incubation period. Samples were rinsed three times in PBS to assess structural abnormalities following a 30-minute staining process with 0.1-μm Alexa-594-Phalloidin to detect actin-containing myofibrils.

*Average Daily Locomotor Activity and Sleep*:

Individual flies were anesthetized with $CO_2$ and placed in glass tubes with food. These tubes were placed in the *Drosophila* Activity Monitor System (DAMS, Trikinetics) (Guo et al. 2024c; Abou Daya et al. 2025). These monitors were placed in incubators to regulate temperature and humidity. The data analysis was performed using Clock lab software, Microsoft Excel, DAM analysis, and Graph Pad Prisml0. Using the Clock lab program, sleep was defined as > 5 minutes of immobility (0 beam breaks over 5 minutes or more). Activity was measured in units of beam breaks, which were collected every 30 seconds. Average activity and sleep were calculated by averaging the total counts over 7 days (Yadav et al. 2025; Abou Daya et al. 2025). Data from the day the individual flies were introduced to the DAMS monitors was not included in the average to allow for adaptation.

### Discussion

Voltage-gated potassium (Kv) channels are fundamental to maintaining electrical excitability in excitable tissues such as the brain and heart. In *Drosophila*, the *Shaker* (*Sh*) gene encodes the alpha subunit of a rapidly inactivating A-type Kv channel that is essential for shaping action potentials and neuronal firing patterns (Sewing et al. 1996). While Shaker channels have been widely investigated in sleep and synaptic transmission, their possible function in regulating heart physiology has received little attention. This is particularly significant because the mammalian homolog Kv1.1 (KCNA1) is present in both the brain and the heart, and its malfunction has been linked to neurological diseases and autonomic cardiac abnormalities (Glasscock et al. 2010; Humphries and Dart 2015). The current discovery identified *Shaker* not only as a neuronal regulator of sleep/activity but also as a crucial molecular link between brain excitability, cardiac output, and circadian-driven behavior, greatly expanding our understanding of its systemic role.

Our findings show that *Shmns* mutant flies, which were previously known for their extremely short-sleeping phenotype (Wu et al. 2008), also exhibit pronounced cardiac dysfunction, such as prolonged heart period, increased arrhythmia, and decreased fractional shortening, all of which are indicators of poor systolic performance (Ma et al. 2020). These deficits were more severe in older flies, indicating a progressive loss in cardiac integrity as reported in aging-associated electrical remodeling described in mammalian hearts with Kv channel impairments (Nattel et al. 2007). This combined disturbance of sleep and cardiac function in *Shmns* mutants reflects what is seen in Kv1.1 knockout mice, which have seizures, impaired sleep, and autonomic cardiac dysregulation (Hu et al. 2025), supporting the notion that Kv channels play an evolutionarily conserved neurocardiac role.

Interestingly, the *Sh*$^{5}$ allele, which affects the same gene, causes milder and age-dependent cardiac abnormalities (Gautam and Tanouye 1990), and trans-heterozygous *Shmns*/*Sh*$^{5}$ flies demonstrate partial recovery of *Shmns*-induced deficits. These findings point to a non-additive, possibly compensating genetic interaction, which may be mediated by mixed tetrameric channel construction or sub-threshold variations in total potassium conductance. This supports studies showing that the composition and stoichiometry of Kv channel subunits can drastically influence gating properties, conductance, and phenotypic severity in both invertebrates and vertebrates (Pongs and Schwarz 2010). We believe such type of allele-specific changes provide valuable genetic tools for exploring channel function under physiological and pathological conditions.

External factors that alter circadian cycles are considered important factors that influence the *Shaker* phenotype. When *Shmns* flies were exposed to continuous light (LL), their cardiac and behavioral parameters degraded, including a decreased heart rate and inadequate sleep. This shows that circadian disruption worsens underlying channelopathies, most likely due to mis regulation of clock-controlled ion channels and cardiac tissue contraction pathways. Circadian genes such *Bmal*1 and *Rev-Erb*α affect Kv channel expression and electrophysiological functions in flies and mammals (Takeda and Maemura 2015). Furthermore, circadian misalignment in cardiac tissue has been shown to enhance sensitivity to arrhythmias, remarkably when repolarization reserves are low (Hayter et al. 2021). Our phalloidin staining indicated altered F-actin containing myofibrillar organization in LL-exposed flies, indicating that light-driven circadian disruption may also interfere with cytoskeletal maintenance, likely through pathways that regulate muscle protein turnover and mitochondrial function (Illescas et al. 2021).

In contrast to light-induced disruption, TRF, intervention, reestablished cardiac function in *Shmns* females by improving the HR, decreasing AI, and shortening the HP to a certain degree. This demonstrates the therapeutic value of feeding cues in altering Kv channel-linked cardiac output. Previous research has shown that TRF improves cardiac mitochondrial efficiency, lowers oxidative stress, and slows cardiac aging in both *Drosophila* and rats (Milan et al. 2024). Remarkably, the TRF benefits were sex-specific in our study, males showed no cardiac rescue, but activity increased, and sleep reduced after TRF (Pataky et al. 2021), which suggest a need to explore how nutrient sensing and hormonal signaling intersect with Kv channel regulation in a sex-dependent manner.

To uncover whether the observed cardiac performance of *Shaker* is cell-autonomous, we implemented tissue-specific knockdowns. Cardiac-specific knockdown using Hand-Gal4 restated many of the *Shmns*

cardiac defects, confirming that *Shaker* functions fundamentally within cardiomyocytes to support contractile performance. This finding is constant with mammalian studies showing that Kv1.5 and Kv1.1 channels contribute to ventricular repolarization and regulation, and that their dysfunction leads to prolonged action potential and electrical instability (Näbauer 1998). Furthermore, neuronal-specific knockdown with Elav-Gal4 disturbed cardiac parameters and decreased sleep, revealed a non-cell-autonomous involvement in control of heart rhythm. The heart in flies gets glutamatergic and neuropeptidergic input, which controls heart rate (Dulcis and Levine 2005b), whereas Kv1.1 failure in autonomic ganglia in mammals causes neurogenic cardiac arrhythmia (Trosclair et al. 2020). These findings emphasize the dual role of central and peripheral *Shaker* activity in cardiac homeostasis and demonstrate how ion channelopathies can cause multisystemic disorders due to common molecular components.

In our study we found that *Shaker* channel as a core integrator of sleep, circadian behavior, and cardiac physiology in *Drosophila*. *Shaker* function links both nervous system and cardiac systems and their sensitivity to environmental cues, including light cycles and feeding patterns. *Shaker* mammalian homologs and its complex role places *Drosophila* in a strong position as a model system to study neurocardiac channelopathies, such as those caused by KCNA1 mutations, long QT syndromes, and sudden unexplained death in epilepsy (SUDEP). Additionally, our results highlight the significance of circadian-aligned interventions, such as TRF, to alleviate channelopathy-induced cardiac dysfunction, suggesting a non-pharmacological approach to restore physiological stability in genetically predisposed systems. Potential research should investigate whether clock genes directly regulate *Shaker* transcription or post-translational modification, and how metabolic signals like AMPK or TOR act together with *Shaker* function across tissues. Understanding these regulatory connections will afford critical insights into how timing, excitability, and metabolism converge at the level of ion channels to shape organismal health.

## Conclusions

This study indicates that the *Drosophila* Shaker potassium channel plays a unique and integrative role in heart physiology, sleep regulation, and circadian behavior. We show that Shaker malfunction causes age-related cardiac function decline and sleep disruption and that these outcomes are influenced by genetic background, environmental cues, and eating behavior. Both cardiac and neuronal knockdowns reveal that Shaker channels contribute to heart function through cell-autonomous and non-cell-autonomous processes, indicating that they are expressed in both the heart and the nervous system. Circadian disturbance worsens *Shaker*-related impairments, but time-restricted eating improves cardiac performance in a sex-dependent manner, emphasizing the role of temporal regulation in ion channel function. These findings support *Shaker*'s role as a crucial molecular link between neurophysiology, circadian regulation, and cardiovascular health, as well as *Drosophila*'s utility as a model for studying neurocardiac channelopathies and designing behavior-based therapeutics. Future research will explore molecular pathways linking *Shaker* dysfunction to cardiometabolic and neurological diseases, aiming to identify therapeutic targets for potassium channelopathies.

## Declarations

**Data availability:** All the raw data are provided as source data.

**Acknowledgement:** We would like to thank Fatma Oduk, researcher in the Melkani lab, for her help with lab management duties, respectively. The Fly stocks were purchased from Bloomington and VDRC.

**Author contribution:** Under the GCM guidelines, DB designed the experiment. DB, AD, SB and KM conducted experiments, analyzed the data, and wrote the draft of the manuscript. KM wrote the manuscript with some initial draft provided by DB and figures. GCM edited and revised the figures and manuscript.

**Funding:** This work was supported by National Institutes of Health (NIH) grants AG065992 and RF1NS133378 to G.C.M. This work is also supported by UAB Startup funds 3,123,226 and 3,123,227 to G.C.M.

All the data are original and have not been published anywhere

**Competing interest:** None

## References

1. Abou Daya F, Mandigo T, Ober L, et al (2025) Identifying links between cardiovascular disease and insomnia by modeling genes from a pleiotropic locus. Dis Model Mech 18:. https://doi.org/10.1242/dmm.052139
2. Bringmann H (2019) Genetic sleep deprivation: using sleep mutants to study sleep functions. EMBO Rep 20:. https://doi.org/10.15252/embr.201846807
3. Bushey D, Huber R, Tononi G, Cirelli C (2007) *Drosophila Hyperkinetic* Mutants Have Reduced Sleep and Impaired Memory. The Journal of Neuroscience 27:5384–5393. https://doi.org/10.1523/JNEUROSCI.0108-07.2007
4. Cammarato A, Ocorr S, Ocorr K (2015) Enhanced Assessment of Contractile Dynamics in *Drosophila* Hearts. Biotechniques 58:77–80. https://doi.org/10.2144/000114255
5. Cirelli C, Bushey D, Hill S, et al (2005a) Reduced sleep in Drosophila Shaker mutants. Nature 434:1087–1092. https://doi.org/10.1038/nature03486
6. Cirelli C, Bushey D, Hill S, et al (2005b) Reduced sleep in Drosophila Shaker mutants. Nature 434:1087–1092. https://doi.org/10.1038/nature03486
7. Cirelli C, Bushey D, Hill S, et al (2005c) Reduced sleep in Drosophila Shaker mutants. Nature 434:1087–1092. https://doi.org/10.1038/nature03486
8. D’Adamo MC, GallenmÃ¼ller C, Servettini I, et al (2015) Novel phenotype associated with a mutation in the KCNA1(Kv1.1) gene. Front Physiol 5:. https://doi.org/10.3389/fphys.2014.00525

9. Dulcis D, Levine RB (2003) Innervation of the heart of the adult fruit fly, *Drosophila melanogaster*. Journal of Comparative Neurology 465:560–578. https://doi.org/10.1002/cne.10869
10. Dulcis D, Levine RB (2005a) Glutamatergic Innervation of the Heart Initiates Retrograde Contractions in Adult *Drosophila melanogaster*. The Journal of Neuroscience 25:271–280. https://doi.org/10.1523/JNEUROSCI.2906-04.2005
11. Dulcis D, Levine RB (2005b) Glutamatergic Innervation of the Heart Initiates Retrograde Contractions in Adult *Drosophila melanogaster*. The Journal of Neuroscience 25:271–280. https://doi.org/10.1523/JNEUROSCI.2906-04.2005
12. Fink M, Callol-Massot C, Chu A, et al (2009a) A New Method for Detection and Quantification of Heartbeat Parameters in Drosophila, Zebrafish, and Embryonic Mouse Hearts. Biotechniques 46:101–113. https://doi.org/10.2144/000113078
13. Fink M, Callol-Massot C, Chu A, et al (2009b) A New Method for Detection and Quantification of Heartbeat Parameters in Drosophila, Zebrafish, and Embryonic Mouse Hearts. Biotechniques 46:101–113. https://doi.org/10.2144/000113078
14. Flourakis M, Kula-Eversole E, Hutchison AL, et al (2015) A Conserved Bicycle Model for Circadian Clock Control of Membrane Excitability. Cell 162:836–848. https://doi.org/10.1016/j.cell.2015.07.036
15. Gautam M, Tanouye MA (1990) Alteration of potassium channel gating: Molecular analysis of the drosophila Sh5 mutation. Neuron 5:67–73. https://doi.org/10.1016/0896-6273(90)90034-D
16. Gill S, Le HD, Melkani GC, Panda S (2015) Time-restricted feeding attenuates age-related cardiac decline in *Drosophila*. Science (1979) 347:1265–1269. https://doi.org/10.1126/science.1256682
17. Glasscock E (2019a) Kv1.1 channel subunits in the control of neurocardiac function. Channels 13:299–307. https://doi.org/10.1080/19336950.2019.1635864
18. Glasscock E (2019b) Kv1.1 channel subunits in the control of neurocardiac function. Channels 13:299–307. https://doi.org/10.1080/19336950.2019.1635864
19. Glasscock E, Yoo JW, Chen TT, et al (2010) Kv1.1 Potassium Channel Deficiency Reveals Brain-Driven Cardiac Dysfunction as a Candidate Mechanism for Sudden Unexplained Death in Epilepsy. The Journal of Neuroscience 30:5167–5175. https://doi.org/10.1523/JNEUROSCI.5591-09.2010
20. Guo Y, Abou Daya F, Le HD, et al (2024a) Diurnal expression of *Dgat2* induced by time-restricted feeding maintains cardiac health in the *Drosophila* model of circadian disruption. Aging Cell 23:. https://doi.org/10.1111/acel.14169
21. Guo Y, Abou Daya F, Le HD, et al (2024b) Diurnal expression of *Dgat2* induced by time-restricted feeding maintains cardiac health in the *Drosophila* model of circadian disruption. Aging Cell 23:. https://doi.org/10.1111/acel.14169
22. Guo Y, Abou Daya F, Le HD, et al (2024c) Diurnal expression of *Dgat2* induced by time-restricted feeding maintains cardiac health in the *Drosophila* model of circadian disruption. Aging Cell 23:. https://doi.org/10.1111/acel.14169

23. Guo Y, Abou Daya F, Le HD, et al (2024d) Diurnal expression of *Dgat2* induced by time-restricted feeding maintains cardiac health in the *Drosophila* model of circadian disruption. Aging Cell 23:. https://doi.org/10.1111/acel.14169
24. Hayter EA, Wehrens SMT, Van Dongen HPA, et al (2021) Distinct circadian mechanisms govern cardiac rhythms and susceptibility to arrhythmia. Nat Commun 12:2472. https://doi.org/10.1038/s41467-021-22788-8
25. Hu A, Aung KP, Reid CA, Soh MS (2025) Kv1.1 channels in cardiorespiratory regulation and sudden unexpected death in epilepsy: insights from mouse models. Brain Commun 7:. https://doi.org/10.1093/braincomms/fcaf116
26. Humphries ESA, Dart C (2015) Neuronal and Cardiovascular Potassium Channels as Therapeutic Drug Targets: Promise and Pitfalls. SLAS Discovery 20:1055–1073. https://doi.org/10.1177/1087057115601677
27. Illescas M, Peñas A, Arenas J, et al (2021) Regulation of Mitochondrial Function by the Actin Cytoskeleton. Front Cell Dev Biol 9:. https://doi.org/10.3389/fcell.2021.795838
28. Kaplan WD, Trout WE (1969) THE BEHAVIOR OF FOUR NEUROLOGICAL MUTANTS OF DROSOPHILA. Genetics 61:399–409. https://doi.org/10.1093/genetics/61.2.399
29. Kim DM, Nimigean CM (2016) Voltage-Gated Potassium Channels: A Structural Examination of Selectivity and Gating. Cold Spring Harb Perspect Biol 8:a029231. https://doi.org/10.1101/cshperspect.a029231
30. Kim J, Ki Y, Lee H, et al (2020) The voltage-gated potassium channel Shaker promotes sleep via thermosensitive GABA transmission. Commun Biol 3:174. https://doi.org/10.1038/s42003-020-0902-8
31. Koh K, Joiner WJ, Wu MN, et al (2008a) Identification of SLEEPLESS, a Sleep-Promoting Factor. Science (1979) 321:372–376. https://doi.org/10.1126/science.1155942
32. Koh K, Joiner WJ, Wu MN, et al (2008b) Identification of SLEEPLESS, a Sleep-Promoting Factor. Science (1979) 321:372–376. https://doi.org/10.1126/science.1155942
33. Ma C, Luo H, Fan L, et al (2020) Heart failure with preserved ejection fraction: an update on pathophysiology, diagnosis, treatment, and prognosis. Brazilian Journal of Medical and Biological Research 53:. https://doi.org/10.1590/1414-431x20209646
34. Milan M, Brown J, O'Reilly CL, et al (2024) Time-restricted feeding improves aortic endothelial relaxation by enhancing mitochondrial function and attenuating oxidative stress in aged mice. Redox Biol 73:103189. https://doi.org/10.1016/j.redox.2024.103189
35. Näbauer M (1998) Potassium channel down-regulation in heart failure. Cardiovasc Res 37:324–334. https://doi.org/10.1016/S0008-6363(97)00274-5
36. Nattel S, Maguy A, Le Bouter S, Yeh Y-H (2007) Arrhythmogenic Ion-Channel Remodeling in the Heart: Heart Failure, Myocardial Infarction, and Atrial Fibrillation. Physiol Rev 87:425–456. https://doi.org/10.1152/physrev.00014.2006

37. Papazian DM, Schwarz TL, Tempel BL, et al (1987) Cloning of Genomic and Complementary DNA from *Shaker* , a Putative Potassium Channel Gene from *Drosophila*. Science (1979) 237:749–753. https://doi.org/10.1126/science.2441470
38. Pataky MW, Young WF, Nair KS (2021) Hormonal and Metabolic Changes of Aging and the Influence of Lifestyle Modifications. Mayo Clin Proc 96:788–814. https://doi.org/10.1016/j.mayocp.2020.07.033
39. Paulhus K, Ammerman L, Glasscock E (2020) Clinical Spectrum of KCNA1 Mutations: New Insights into Episodic Ataxia and Epilepsy Comorbidity. Int J Mol Sci 21:2802. https://doi.org/10.3390/ijms21082802
40. Pongs O, Kecskemethy N, Müller R, et al (1988) Shaker encodes a family of putative potassium channel proteins in the nervous system of Drosophila. EMBO J 7:1087–1096. https://doi.org/10.1002/j.1460-2075.1988.tb02917.x
41. Pongs O, Schwarz JR (2010) Ancillary Subunits Associated With Voltage-Dependent $K^+$ Channels. Physiol Rev 90:755–796. https://doi.org/10.1152/physrev.00020.2009
42. Sewing S, Roeper J, Pongs O (1996) Kvβ1 Subunit Binding Specific for Shaker-Related Potassium Channel α Subunits. Neuron 16:455–463. https://doi.org/10.1016/S0896-6273(00)80063-X
43. Takeda N, Maemura K (2015) The role of clock genes and circadian rhythm in the development of cardiovascular diseases. Cellular and Molecular Life Sciences 72:3225–3234. https://doi.org/10.1007/s00018-015-1923-1
44. Tempel BL, Jan YN, Jan LY (1988) Cloning of a probable potassium channel gene from mouse brain. Nature 332:837–839. https://doi.org/10.1038/332837a0
45. Tempel BL, Papazian DM, Schwarz TL, et al (1987) Sequence of a Probable Potassium Channel Component Encoded at *Shaker* Locus of *Drosophila*. Science (1979) 237:770–775. https://doi.org/10.1126/science.2441471
46. Trosclair K, Dhaibar HA, Gautier NM, et al (2020) Neuron-specific Kv1.1 deficiency is sufficient to cause epilepsy, premature death, and cardiorespiratory dysregulation. Neurobiol Dis 137:104759. https://doi.org/10.1016/j.nbd.2020.104759
47. Ueda A, Wu C-F (2006) Distinct Frequency-Dependent Regulation of Nerve Terminal Excitability and Synaptic Transmission by IA and IK Potassium Channels Revealed by Drosophila Shaker and Shab Mutations. Journal of Neuroscience 26:6238–6248. https://doi.org/10.1523/JNEUROSCI.0862-06.2006
48. Villanueva JE, Livelo C, Trujillo AS, et al (2019) Time-restricted feeding restores muscle function in Drosophila models of obesity and circadian-rhythm disruption. Nat Commun 10:2700. https://doi.org/10.1038/s41467-019-10563-9
49. Whicher JR, MacKinnon R (2016) Structure of the voltage-gated $K^+$ channel Eag1 reveals an alternative voltage sensing mechanism. Science (1979) 353:664–669. https://doi.org/10.1126/science.aaf8070

50. Wu MN, Koh K, Yue Z, et al (2008) A Genetic Screen for Sleep and Circadian Mutants Reveals Mechanisms Underlying Regulation of Sleep in Drosophila. Sleep 31:465–472. https://doi.org/10.1093/sleep/31.4.465
51. Yadav A, Ouyang X, Barkley M, et al (2025) Regulation of lipid dysmetabolism and neuroinflammation linked with Alzheimer's disease through modulation of Dgat2

## Figures

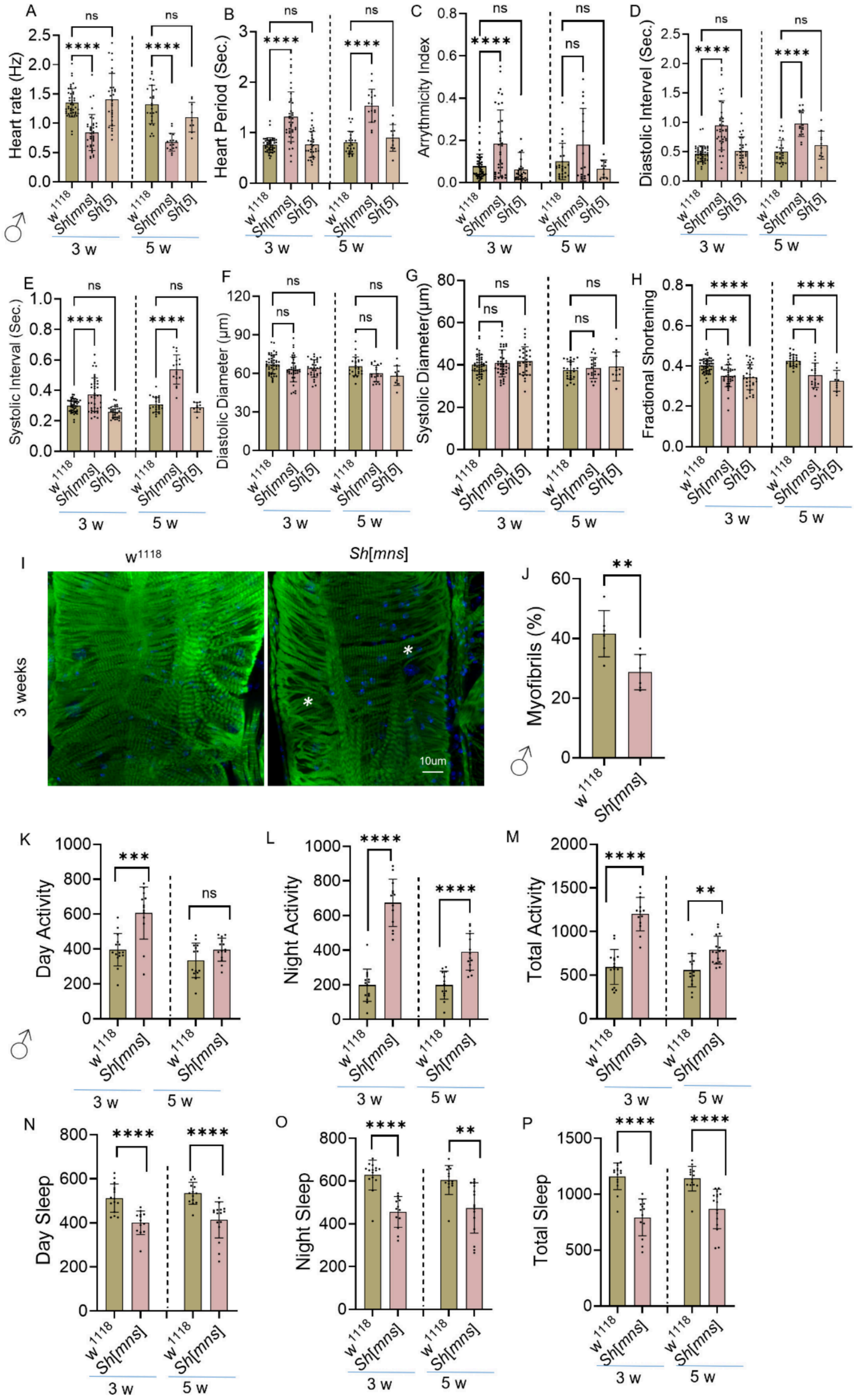

### Figure 1

*Sh*$^{mns}$ and *Sh*5 mutant alleles inversely impact heart function: The heart physiology and sleep/activity of three-week and five-week-old male flies were analyzed for the *Shaker* mutants mini-sleep (*Shmns*) and 5 (*Sh*5) and compared to that of the age and sex-matched control line w$^{1118}$. Different variables of heart function were measured and represented in individual graphs: (A-H) represent data collected from threeweek and five-week-old male *Shmns* $w^{1118}$ and *Sh*5 flies. Phalloidin staining to show myofibril percentage in heart muscle, measured using Image J. (K-P) sleep/activity analysis $w^{1118}$ and *Shmns* at 3 weeks and 5 weeks of age. Statistics: Heart physiology and sleep/activity analysis performed by One-Way ANOVA. Myofibril percent was calculated based on Image J using an Unpaired t-test. $p>0.05$ (ns), $p<0.02$ (*), $p<0.0002$ (***), $p<0.0001$(****).

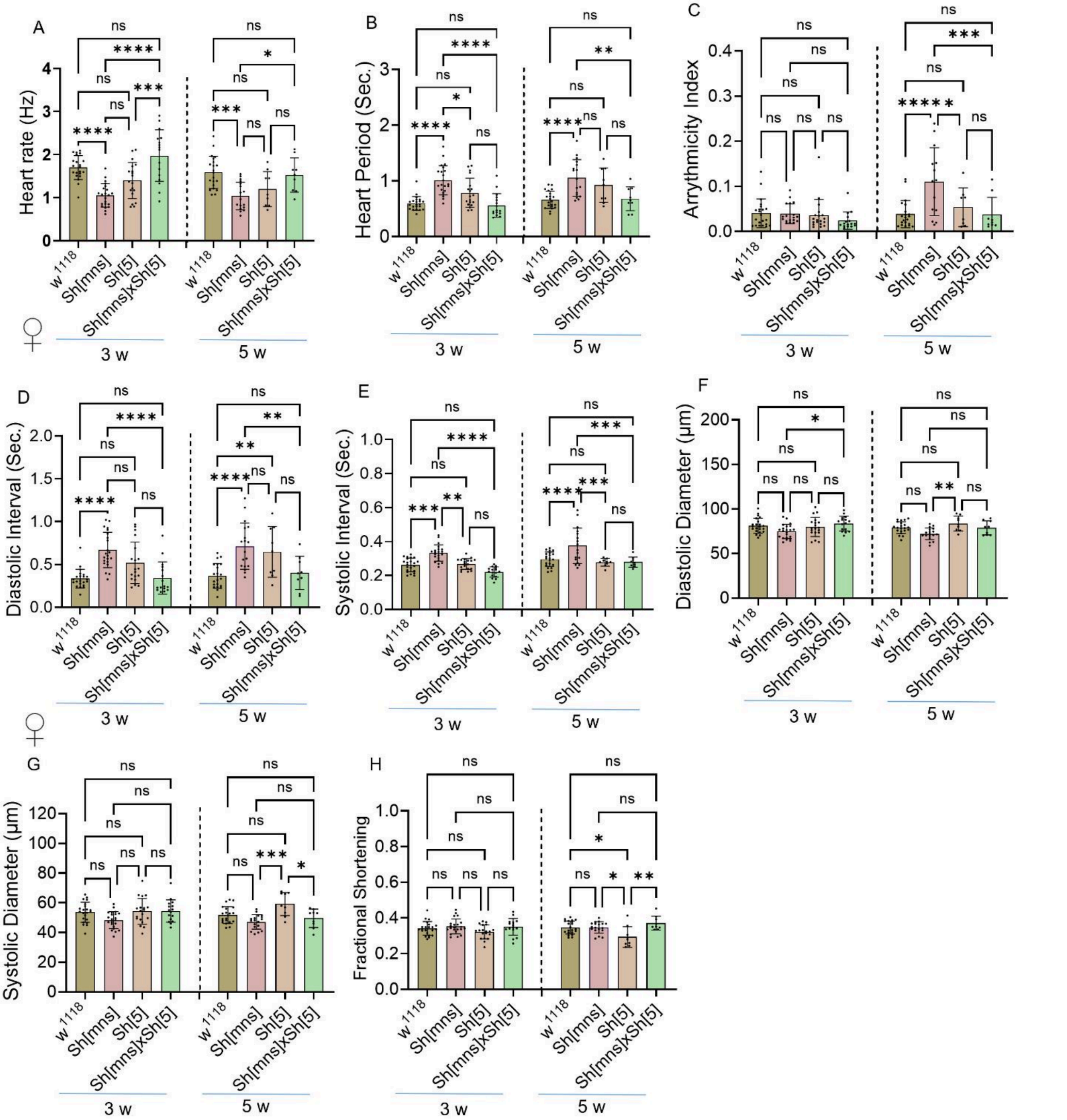

### Figure 2

Significance of *Shaker* mutants on female heart function: (A-H) Represents heart physiology analysis data collected from three and five-week-old females (A-H). The w1118, *Shmns*, *Sh*5 and *Shmns*x *Sh*5 flies. Statistics: Heart physiology analysis performed by Two-Way ANOVA. p>0.05 (ns), p<0.05 (*), p<0.002 (**), p<0.0002 (***), and p<0.0001 (****).

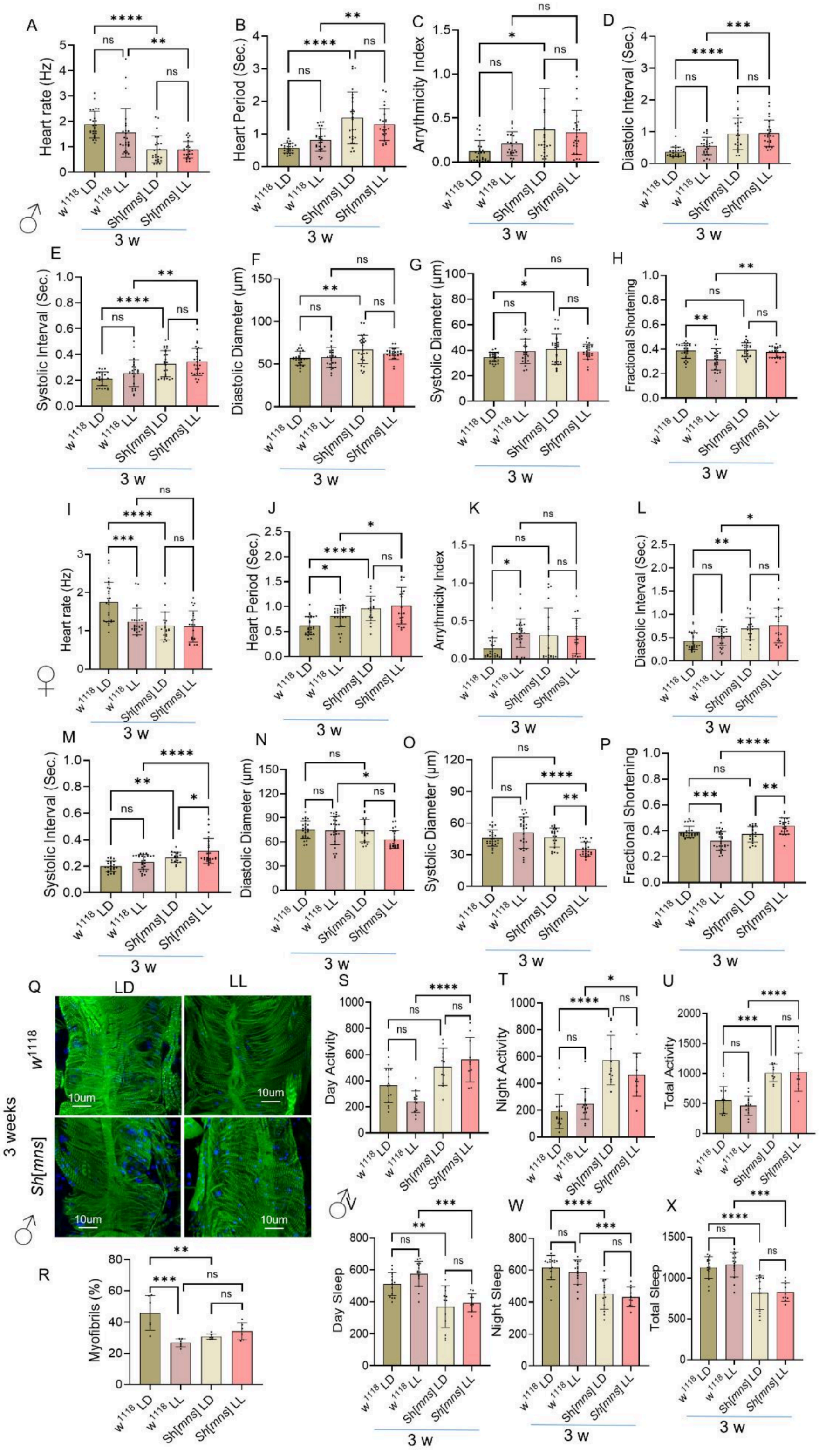

**Figure 3**

Role of external cues (Light-Dark and Light-Light cycles) on *Shmns* heart physiology and sleep/activity: Represents the impact of light/dark (LD) and light/light (LL) cues-induced circadian cycle disruption on male heart physiology (A-H) and Female heart physiology under LL, LD cycles represent (I-P). Myofibril percentage in LD and LL $w^{1118}$, *Shmns* genotypes (Q, R). Sleep/activity patterns of three-week-old flies (S-X). Statistics: Heart physiology, phalloidin staining and sleep/activity analysis performed by Two-Way ANOVA. p<0.05 (*), p<0.002 (**), p<0.0002 (***), p<0.0001(****).

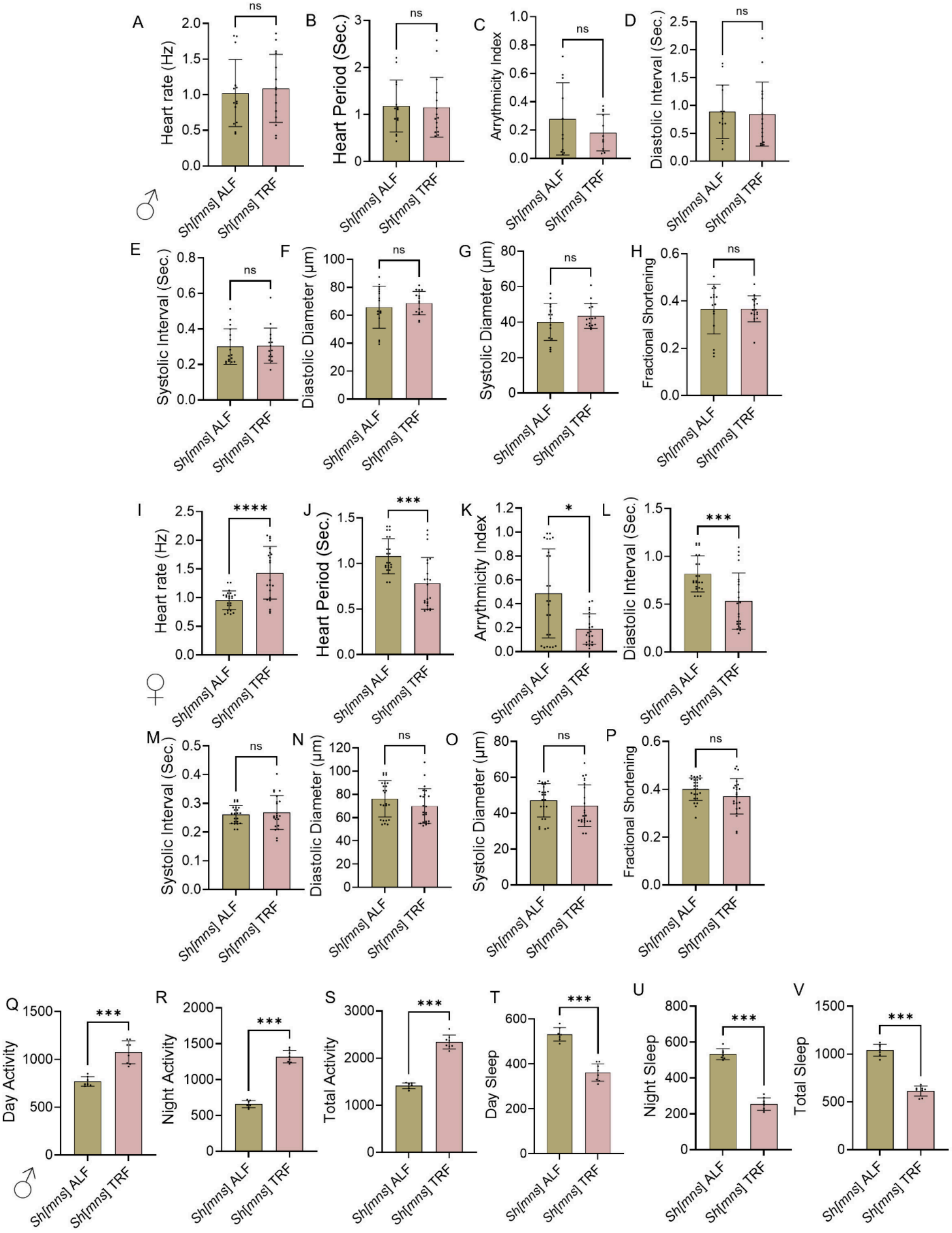

Figure 4

Impact of feeding times on heart physiology and sleep/activity in *Shmns* flies: Represents the impact of the availability of feeding times on heart function (A-H) in males and (I-P) in female flies at 3 weeks of age. Sleep/activity in male flies (Q-S). Statistics: Heart physiology, and sleep/activity analysis performed by Unpaired t test p>0.05 (ns), p<0.002 (**), p<0.0002 (***).

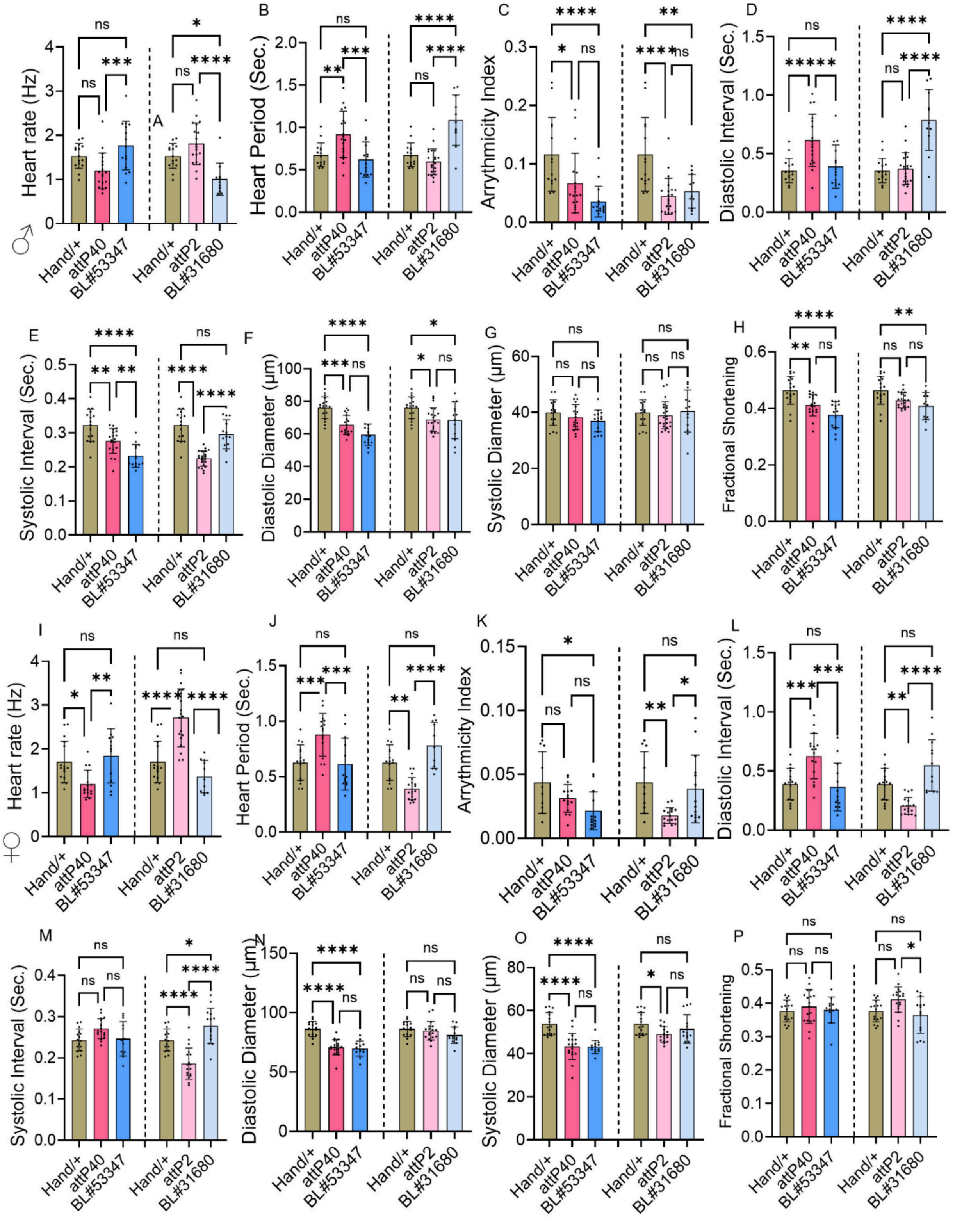

**Figure 5**

Cardiac specific expression of *Shaker* gene impacting heart function: Represents the cardiac specific expression of *Shaker* genes and its knockdown phenotypes in males at 3 weeks (A-H) and female (I-P) 3 weeks flies studied here we crossed cardiac specific driver (Hand-Gal4) with $w^{1118}$, *Shaker* KD (BL#53347, BL#31680) genes and empty vectors for 2nd (attp40) and 3rd (attP2) chromosomes as internal controls. Statistics: Heart physiology analysis performed by One-Way ANOVA. p<0.05 (*), p<0.002 (**), p<0.0002 (***), p<0.0001 (****).

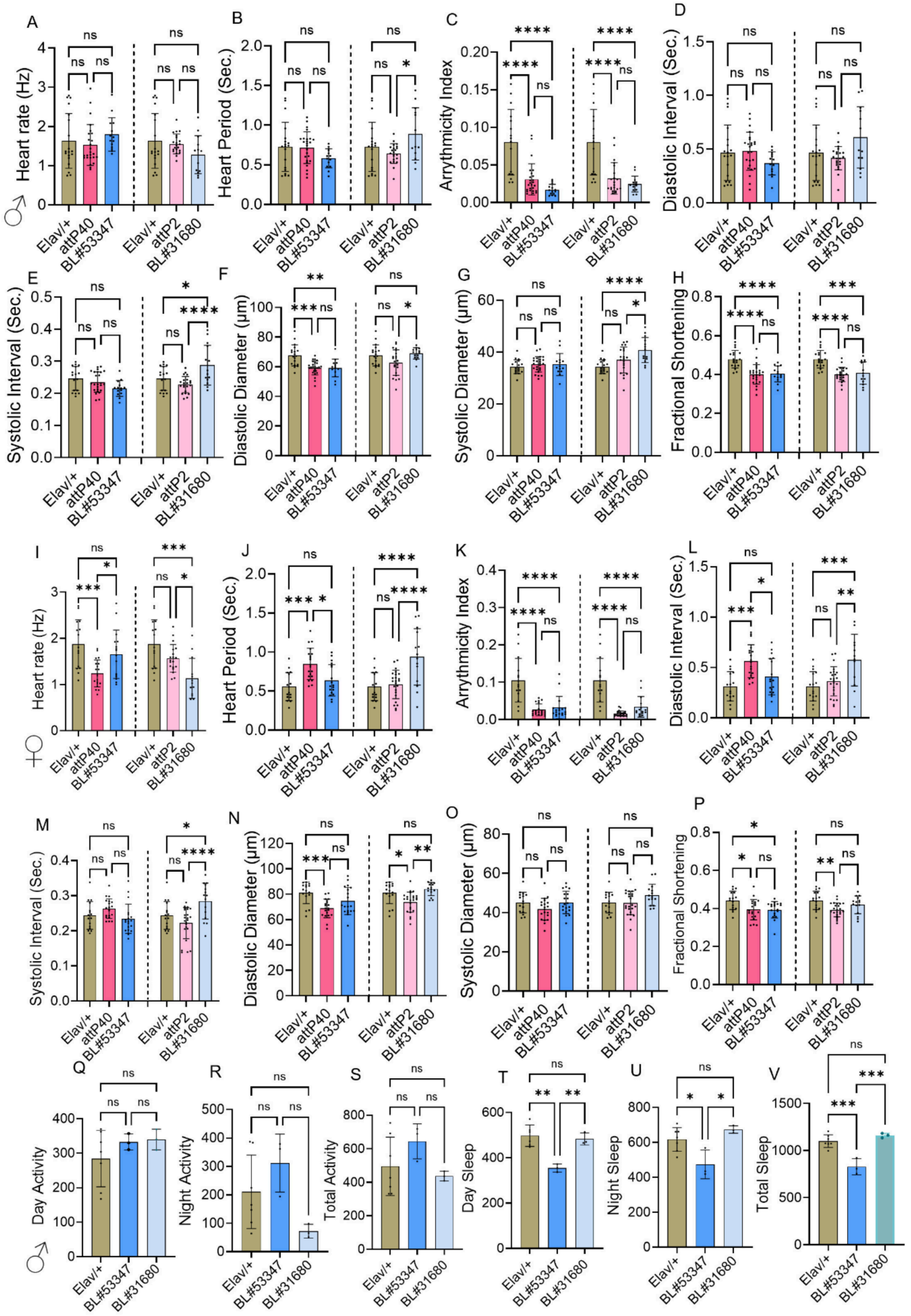

**Figure 6**

Pan-neuronal expression of *Shaker* gene influencing heart function: Represents the neuronal specific expression of *Shaker* genes and its knockdown phenotypes in males at 3 weeks (A-H) and female (I-P) 3 weeks flies studied here we crossed pan-neuronal specific driver (Elav-Gal4) with $w^{1118}$, *Shaker* KD (BL#53347, BL#31680) genes and empty vectors for 2nd (attp40) and 3rd (attP2) chromosomes as internal controls. (Q-V) Sleep/activity analysis at 3 weeks of age. Statistics: Heart physiology analysis performed by One-Way ANOVA. $p<0.05$ (*), $p<0.002$ (**), $p<0.0002$ (***), $p<0.0001$ (****).

## Supplementary Files

This is a list of supplementary files associated with this preprint. Click to download.

- Onlinefloatimage1.png
- Summaryfigure.docx
